# All-*trans* retinoic acid induces lipophagy by reducing Rubicon in Hepa1c1c7 cells

**DOI:** 10.1016/j.jlr.2024.100598

**Published:** 2024-07-18

**Authors:** Anh The Nguyen, Masashi Masuda, Yuki Mori, Yuichiro Adachi, Teppei Fukuda, Airi Furuichi, Masaki Takikawa, Yuki Tsuda, Yuki Hamada, Yusuke Maruyama, Hirokazu Ohminami, Kohta Ohnishi, Yutaka Taketani

**Affiliations:** Department of Clinical Nutrition and Food Management, Institute of Biomedical Sciences, Tokushima University Graduate School, Tokushma, Tokushima, Japan

**Keywords:** lipid droplets, lipolysis and fatty acid metabolism, liver, nutrition, vitamin A

## Abstract

All-*trans* retinoic acid (atRA), a metabolite of vitamin A, reduces hepatic lipid accumulation in liver steatosis model animals. Lipophagy, a new lipolysis pathway, degrades a lipid droplet (LD) via autophagy in adipose tissue and the liver. We recently found that atRA induces lipophagy in adipocytes. However, it remains unclear whether atRA induces lipophagy in hepatocytes. In this study, we investigated the effects of atRA on lipophagy in Hepa1c1c7 cells and the liver of mice fed a high-fat diet (HFD). First, we confirmed that atRA induced autophagy in Hepa1c1c7 cells by Western blotting and the GFP-LC3-mCherry probe. Next, we evaluated the lipolysis in fatty Hepa1c1c7 cells treated with the knockdown of *Atg5*, an essential gene in autophagy induction. *Atg5*-knockdown partly suppressed the atRA-induced lipolysis in fatty Hepa1c1c7 cells. We also found that atRA reduced the protein, but not mRNA, expression of Rubicon, a negative regulator of autophagy, in Hepa1c1c7 cells and the liver of HFD-fed mice. *Rubicon*-knockdown partly inhibited the atRA-induced lipolysis in fatty Hepa1c1c7 cells. In addition, atRA reduced hepatic Rubicon expression in young mice, but the effect of atRA on it diminished in aged mice. Finally, we investigated the mechanism underlying reduced Rubicon protein expression by atRA in hepatocytes. A protein synthesis inhibitor, but not proteasome or lysosomal inhibitors, significantly blocked the reduction of Rubicon protein expression by atRA in Hepa1c1c7 cells. These results suggest that atRA may promote lipophagy in fatty hepatocytes by reducing hepatic Rubicon expression via inhibiting protein synthesis.

Non-alcoholic fatty liver disease (NAFLD), defined by the abundance of lipid droplets (LDs) in hepatocytes, is the leading cause of chronic liver disease globally, which is estimated to affect approximately 25% of the world’s adult population (1). NAFLD can comprise simple steatosis or more advanced non-alcoholic steatohepatitis (NASH), which is characterized by inflammation and hepatocyte injury that is often accompanied by fibrosis. While NAFLD can occur as a consequence of diseases such as obesity and type II diabetes, NAFLD increases the risk of type II diabetes, dyslipidemia, hypertension, cardiovascular disease, chronic kidney disease, liver cirrhosis, and hepatocellular carcinoma (1, 2). Although simple steatosis can be reversible, NASH can progress to liver cirrhosis, hepatocellular carcinoma, and mortality (1, 2).

Autophagy maintains cellular homeostasis by targeting proteins and damaged organelles (3). An isolation membrane encloses a portion of cytoplasm, forming a characteristic double-membraned organelle termed the autophagosome. The autophagosome then fuses with the lysosome to form an autolysosome, which is then degraded by lysosomal enzymes (4, 5). During autophagy, LC3 is conjugated to the phosphatidylethanolamine molecule on the isolation membrane as lipidated LC3 (LC3-II), an autophagy substrate, by the ATG12-ATG5-ATG16L1 complex after cleavage of glycine 120 residues of LC3 by ATG4, and LC3-II contributes to the formation of the isolation membrane (3, 4, 5). Autophagy can enhance lipid catabolism and decrease hepatic steatosis, a phenotype of NAFLD, via lipophagy, a lipolysis pathway that degrades LD via autophagy (1, 6, 7, 8). Since autophagy/lipophagy is impaired in NAFLD, these studies highlight the potential of promoting lipophagy as a therapeutic approach to treat NAFLD (1, 8, 9). In addition, some evidence suggests that the incidence of NAFLD is steadily increasing in the elderly population and aged animals (10). An age-dependent decline in basal autophagy in the liver may underlie the accumulation of hepatic lipids, contributing to a vicious cycle promoting aging (11, 12). Generally, autophagy can be induced in cells through the activation of AMP-activated protein kinase (AMPK) or the inhibition of the mammalian target of rapamycin complex 1 (mTORC1) (13, 14). On the other hand, Rubicon, an autophagy repressor, is localized at lysosomes and suppresses autophagy at the autophagosome-lysosome fusion step via interacting with UVRAG and Rab7 (15, 16, 17). Interestingly, recent studies revealed that Rubicon protein expression increases in the aged livers of mice but decreases in the aged adipose of mice (18, 19). In addition, increased Rubicon causes decreased autophagic activity in the liver of mice fed a high-fat diet (HFD) (20). Therefore, Rubicon may become a valuable therapeutic target for promoting hepatic autophagy in NAFLD.

All-trans retinoic acid (atRA) is an active metabolite of vitamin A, which plays a vital role in cell growth, differentiation, apoptosis, muscle fiber type, and other cell functions (21, 22, 23). Some studies revealed that atRA exerts anticancer effects on the liver by inhibiting the proliferation of liver cancer cells (24, 25), and atRA promotes autophagy in hepatocellular carcinoma cell lines and liver with ischemia and reperfusion in mice (26, 27). atRA has also been reported to reduce hepatic lipid accumulation in liver steatosis model animals by repressing peroxisome proliferator-activated receptor gamma (PPARγ) and to induce lipolysis by a PPARβ/δ-mediated increase in the levels of hormone-sensitive lipase (HSL) in adipocytes (28, 29). Recently, we found that atRA induces lipophagy by activating the AMPK pathway in adipocytes. Interestingly, although we also demonstrated that atRA reduces Rubicon protein expression in adipocytes, we have not determined whether Rubicon is associated with the induction of lipophagy by atRA in adipocytes (30). In addition, it remains unclear whether atRA ameliorates hepatic steatosis by inducing lipophagy and whether Rubicon is involved in its molecular mechanism.

In the present study, we investigated the effects of atRA on hepatic lipophagy and Rubicon expression in mice fed an HFD or aged mice and in Hepa1c1c7 cells. Besides, we also elucidated the role of Rubicon on atRA-induced lipophagy in fatty acid-induced lipid deposition in Hepa1c1c7 cells and the part of the mechanism of reducing Rubicon expression by atRA.

## Materials and Methods

### Chemicals and reagents

DMEM high glucose (08458-16), penicillin-streptomycin, Chemi-Lumi One Super, BSA, FFA-free BSA, and 4′,6-diamidino-2-phenylindole (DAPI; D9542) were purchased from Nacalai Tesque. Torin 1 was purchased from Cayman Chemical Co.. TransIT-LT1 Reagent was purchased from TaKaRa. DMSO, atRA, palmitic acid, oleic acid, mouse anti-β-actin monoclonal Ab (A5441), FBS, AGN193109, Mission siRNA oligos, and cycloheximide were purchased from Sigma-Aldrich. RIPA buffer, anti-LC3B (#2775), anti-p62 (#5114), anti-GFP (#2912), and anti-Rubicon (#8465) Ab were purchased from Cell Signaling Technology. Goat anti-rabbit IgG (H + L)-HRP conjugate (#1706515) was purchased from Bio-Rad. Goat anti-mouse IgG (H + L)-HRP conjugate (#62-6520) and Alexa Fluor 488 were purchased from invitrogen. Anti-mCherry (ab167453) was purchased from Abcam. 4-[E−2-(5, 6, 7, 8-Tetrahydro-5, 5, 8, 8-tetra-methyl-2-naphtalenyl)-1-propenyl] benzoic acid (TTNPB) was purchased from Biomol Research Laboratories. Anti-Rubicon (21444-1-AP) Ab was purchased from Proteintech. Aqua-Poly/Mount (18606-20) was purchased from Polysciences, Inc. Buprenorphine hydrochloride was purchased from Otsuka Pharmaceutical Co., Ltd.. Pentobarbital sodium salt was purchased from Tokyo Kasei Co., Ltd.. Bafilomycin A1 was obtained from Enzo Life Science. FluoroBrite™ DMEM (A1896701), Lipofectamine RNAiMAX transfection reagent, TRIzol™ Reagent, oligo(dT) primer, and Fast SYBR^Ⓡ^ Green master mix were purchased from Thermo Fisher Scientific. M-MLV reverse transcriptase was purchased from Nippon Gene.

### Cell culture

The murine hepatoma cell line, Hepa1c1c7 (CRL-2026) was obtained from ATCC. Hepa1c1c7 cells were cultured in high-glucose DMEM containing 10% FBS, 100 units/ml penicillin, and 100 μg/ml streptomycin at 37°C in a humidified atmosphere of 5% CO_2_. Cells were allowed to grow in 10-cm dish for 2 days and were transferred to new culture media at ∼80% confluence. To induce the lipid accumulation in vitro (fatty Hepa1c1c7 cells), Hepa1c1c7 cells were incubated with 1 mM FFA mixture comprising fatty acid-free BSA-conjugated oleic and palmitic acid in the ratio 2:1 for 4 days. In all experiments, cells were treated with reagents at 100% confluence.

### Oil red-O staining

To determine the triglycerides accumulated in Hepa1d1c7 cells, oil red-O staining was performed using 12-well culture plates. Cells were cultured to 100% confluence and then treated with 1 mM of the mixture of oleic acid and palmitic acid (ratio 2:1) for 4 days to induce lipid accumulation. Then cells were treated with the vehicle (DMSO) or atRA (100 nM) for 14 days. After removing the medium and washing twice with PBS, the cells were fixed with 4% PFA/PBS for 10 min. After washing with PBS, 60% isopropanol was added for 1 min and stained with oil red-O diluted with 60% isopropanol for 20 min. After rinsing with 60% isopropanol and PBS, the cells were photographed under a BZ-X800 fluorescence microscope. Lipid area size was quantified using the ImageJ imaging software program.

### Western blot analysis

Tissue and cell lysates were prepared using RIPA buffer. Protein samples were heated at 95°C for 5 min in sample buffer in the presence of 5% 2-mercaptoethanol and subjected to SDS-PAGE. The separated proteins were transferred by electrophoresis to polyvinylidene difluoride transfer membranes (Immobilon-P, Millipore). The membranes were treated with diluted affinity-purified anti-LC3B (1:2,000), anti-p62 (1:2,000), and anti-Rubicon (1:2,000) Ab. Mouse anti-β-actin (1:4,000) monoclonal Ab was used as an internal control. Goat anti-rabbit IgG(H + L)-HRP conjugate or goat anti-mouse IgG(H + L)-HRP conjugate was utilized as a secondary Ab, and signals were detected using Chemi-Lumi One Super.

### Plasmid construction and establishment of stable cell lines

Retroviral plasmid pMRX-IP-GFP-LC3-mCherry to make more sensitive detection for autophagy flux assay than pMRX-IP-GFP-LC3-RFP-LC3ΔG was generated as follows: Retroviral plasmid vector of pBABE-puro mCherry-EGFP-LC3 (#22418) developed by Dr Jayante Debnath was obtained from Addgene (31). The retroviral plasmid vector of pMRX-IP-GFP-LC3-RFP-LC3ΔG (RDB14600) developed by Dr. Noboru Mizushima was obtained from RIKEN BRC DNA BANK (32). A DNA fragment of encoding mCherry was amplified by RT-PCR with pBABE-puro mCherry-EGFP-LC3 as a template using primers (forward: 5′-GATCTACGCGTATGGTGAGCAAGG-3′; reverse: 5′-GGATCTGGATCCTCACTTGTACAGC-3′). Then, the PCR product was inserted into the pMRX-IP-GFP-LC3 vector which was amplified by RT-PCR with pMRX-IP-GFP-LC3-RFP-LC3ΔG as a template to delete the DNA fragment of RFP-LC3ΔG using primers (forward: TGAAACGCGTGATGACGTCCTCG; reverse: CTAAGGATCCCAGTGTGGTGGTACG-3′).

This plasmid was transfected into 50% confluent Hepa1c1c7 cells using TransIT-LT1 Reagent for 48 h. The medium was replaced with a medium containing the final concentration of 2 μg/ml puromycin for 10 days. The cells were then subjected to single-cell sorting using a JSAN cell sorter (Bay Bioscience) to yield a single clone that strongly emitted both GFP and mCherry fluorescence. The FL1 filter (λ ex: 488 nm, λ em: 525 nm) and FL7 (λ ex: 586 nm, λ em: 600 nm) were used for the detection of GFP and mCherry, respectively.

### Autophagy flux assay

For GFP-LC3-mCherry microplate assay, wild-type (as background) or stable Hepa1c1c7 cells expressing GFP-LC3-mCherry were seeded into 96-well plates (Greiner CELLSTAR #655090, Greiner Bio-One). Cells were allowed to grow up to 100% confluence, then they were treated with vehicle (DMSO) or 100 nM atRA for 24 h. Following washing with FluoroBrite DMEM twice, cells were imaged by using Operetta high-content imaging system (PerkinElmer) at 40x magnification at following settings: for EGFP (λ ex: 460−490 nm, λ em: 500−550 nm) and for mCherry (λ ex: 530−560 nm, λ em: 570−650 nm).

### RNAi experiments

Hepa1c1c7 cells were transfected with siRNA directed against *Atg5* (SASI_Mm01_00089196 and SASI_Mm01_00089197; Sigma-Aldrich), *Rubicon* (SASI_Mm02_00316863; Sigma-Aldrich), or negative control (SIC001; Sigma-Aldrich) using Lipofectamine RNAiMAX transfection reagent, according to the manufacture's instruction.

### Detection of NEFA in medium

NEFA content in the medium was determined as described previously (33). To determine the release of fatty acids from LD in differentiated Hepa1c1c7 cells, the detection of NEFA in the medium was performed using 48-well tissue culture plates. Cells were treated with a 1 mM mixture of FFA to induce lipid accumulation and transfection with 10 pmol *Atg5* siRNA, 10 pmol *Rubicon*, or siControl for 24 h, followed by replacement 2% FFA-free BSA culture medium and the treatment with vehicle (DMSO) or 100 nM atRA for 48 h. After collecting the medium, NEFA content in the conditioned medium was measured with a commercial kit LabAssay NEFA (Wako) according to the manufacturer's protocol. The sample was mixed with reagent 1 and incubated at 37°C for 10 min. Then, reagent 2 was added and after 10 min incubation at 37°C, a colored product was formed with a maximal absorbance at 550 nm. The data were calibrated using the standard curve.

### Quantitative PCR analysis

Total RNA was isolated from Hepa1c1c7 cells or liver tissues using TRIzol™ Reagent according to the manufacturer's instructions. Quantitative real-time PCR assays were performed using an Applied Biosystems StepOne qPCR instrument. In brief, the cDNA was synthesized from 1 μg of total RNA using M-MLV reverse transcriptase with an oligo(dT) primer. After cDNA synthesis, quantitative real-time qPCR was performed in 5 μl of Fast SYBR^Ⓡ^ Green PCR master mix. The primer sequences (mouse *Rubicon* and mouse *β-actin*) were described previously (33).

### Immunofluorescence staining

Hepa1c1c7 cells were cultured to 100% confluence on coated glasses and then fixed with 3% PFA for 15 min at room temperature (RT) followed by permeabilization with 0.1% Triton X-100 for 10 min and blocking with 1% BSA for 30 min. Cells then were incubated overnight at 4˚C with anti-Rubicon antibody. Then cells were incubated at RT with green fluorescent anti-rabbit IgG and DAPI solution for 1 h. Then, cells were washed 3 times with PBS and mounting. After drying, image acquisition was at 100x oil magnification using BZ-X800 fluorescence microscope. Image quantification was performed using image analysis software by measuring the intensity of green areas where the Rubicon is expressed. A minimum of 5 image fields were used for the analysis in each group.

### Animal experiments

The animal work took place in the Division for Animal Research and Genetic Engineering Support Center for Advanced Medical Sciences, Institute of Biomedical Sciences, Tokushima University Graduate School. The animals were housed in pathogen-free conditions and maintained under a standard 12 h light-dark cycle with free access to water. First, seven-week-old male C57BL/6J mice (Japan SLC, Shizuoka, Japan) were fed an HFD, that contained 45% kcal as fat, 35% kcal as carbohydrate, 20% kcal as a protein with an energy density of 4.73 kcal/gm (No. D12451; Research Diets, New Brunswick) for 8 weeks. Then mice were randomly divided into two groups (n = 5 per group) and intraperitoneally administrated a total of 10 mg/kg body weight (BW) of atRA or 1% DMSO prepared in 500 μl sterile saline once every two days for 4 weeks. Secondly, young (8-week-old) and aged (86-week-old) mice were randomly divided into two groups each (n = 5 per group) and a total of 10 mg/kg body weight of atRA or 1% DMSO (control) prepared in sterile saline was intraperitoneally administered, then the mice were euthanized 24 h later. Each group of mice was fasted for 20 h with water ad libitum before sacrifice with a total of 0.1 mg/kg BW of buprenorphine hydrochloride and a total of 50 mg/kg BW of pentobarbital sodium salt, and tissues were removed. Liver samples were washed in 0.9% NaCl and immediately snap-frozen in liquid nitrogen and stored at −80°C. These studies were approved by the Animal Experimentation Committee of Tokushima University School of Medicine (animal ethical clearance No. T28-88 and T30-66) and were carried out in accordance with guidelines for the Animal Care and Use Committee of Tokushima University School of Medicine.

### Statistical analysis

Data were collected from more than two independent experiments and were reported as the mean and SEM. The statistical analysis for the two-group comparison was performed using an unpaired two-tailed *t* test. All data analyses were performed using the GraphPad Prism 5 software program (GraphPad Software). *P* values of <0.05 were considered to indicate statistical significance.

## Results

### atRA partially contributes to lipolysis through autophagy in Hepa1c1c7 cells

**Fig. 1:**
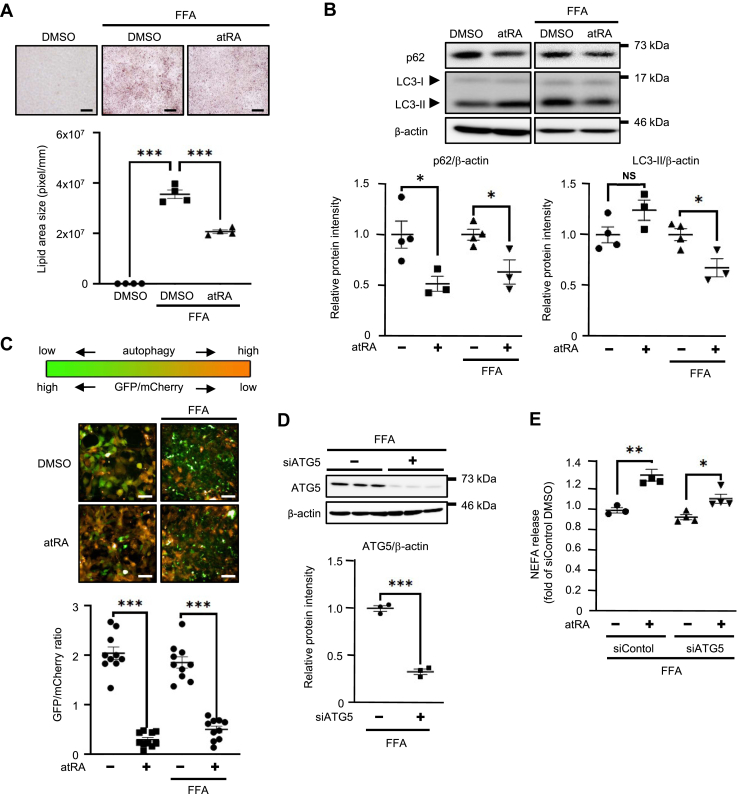
Effects of atRA on the lipolysis of LD through autophagy in Hepa1c1c7 cells. A: Hepa1c1c7 cells were treated with 1 mM mixture of oleic acid and palmitic acid (ratio 2:1) for 4 days as an FFA-treated group to induce lipid accumulation (fatty Hepa1c1c7 cells), then cells were treated with 100 nM atRA or 0.1% DMSO (vehicle) for 14 days. Cells were then fixed, and stained with oil red-O staining. Scale bar = 100 μm. Lipid accumulation was quantified using the ImageJ imaging software program (n = 4). B: Western blotting of LC3 and p62 proteins in Hepa1c1c7 or fatty Hepa1c1c7 cells treated with atRA or DMSO (vehicle) for 24 h (n = 3–4). β-actin was used as an internal control. C: autophagic flux in Hepa1c1c7 or fatty Hepa1c1c7 cells stably expressing GFP-LC3-mCherry treated with atRA or DMSO for 24 h. GFP/mCherry ratio data were expressed as the fold-value against DMSO (n = 10). Images were taken with a fluorescence microscope. Scale bar = 50 μm. D: endogenous ATG5 protein was detected by western blotting 48 h after transfection with 100 pmol *Atg5* siRNA (siATG5) or siControl (n = 3). ∗∗∗*P* < 0.001 (two-tailed unpaired *t* test). E: Fatty Hepa1c1c7 cells were treated with atRA or DMSO in DMEM containing 2% BSA-FFA for 48 h after transfection with siATG5 or siControl (n = 3–4). The culture medium was collected and assayed for NEFA content (mEq/L/mg protein). Values are mean ± SEM. ∗*P* < 0.05, ∗∗*P* < 0.01, ∗∗∗*P* < 0.001 (one-way ANOVA with a Student-Newman *post hoc* test). NS, not significant.

Although Hepa1c1c7 cells are mouse hepatoma cell lines, Hepa1c1c7 cells have been used to study hepatocyte lipid accumulation (34, 35, 36). Therefore, we believe that there is no problem in using Hepa1c1c7 cells in our experiment. To investigate the effect of atRA on lipolysis in hepatocytes, we first treated Hepa1c1c7 cells with a mixture of FFA for 4 days to induce fatty Hepa1c1c7 cells, then cells were treated with 0.1% DMSO (vehicle) or 100 nM atRA for 14 days. Oil red-O staining showed the lipid accumulation remarkably decreased by atRA compared to DMSO treatment in fatty Hepa1c1c7 cells (Fig. 1A). We next investigated whether atRA affects autophagy-related protein expression in Hepa1c1c7 cells. Western blotting showed that the expression of LC3-II did not increase after 24 h of treatment with atRA in Hepa1c1c7 or fatty Hepa1c1c7 cells. On the other hand, atRA treatment decreased the expression of p62, a cargo receptor for autophagic degradation, in Hepa1c1c7 or fatty Hepa1c1c7 cells (Fig. 1B). As a novel methodology for measuring autophagy flux, the GFP-LC3-RFP-LC3ΔG and GFP-LC3-RFP probe were established (32). A decline in the GFP/RFP ratio indicates enhanced autophagy. We have previously reported that sulforaphane or atRA decreased the GFP/RFP ratio in differentiated 3T3-L1 adipocytes stably expressing the GFP-LC3-RFP-LC3ΔG (33). In this study, we created another probe, GFP-LC3-mCherry (Supplemental Fig. S1A), to construct an autophagy activity evaluation system with higher detection sensitivity because the brightness of the red fluorescence of mCherry is higher than that of RFP (37). Firstly, we determined whether Hepa1c1c7 cells stably expressing the GFP-LC3-mCherry are suitable for the autophagy flux assay by autophagy inducer Torin 1 or autophagy inhibitor Bafilomycin A1. As a result, we confirmed that Hepa1c1c7 cells stably expressing the GFP-LC3-mCherry functioned correctly (Supplemental Fig. S1B, C). In Hepa1c1c7 cells stably expressing the GFP-LC3-mCherry, atRA treatment significantly decreased the GFP/mCherry ratio compared with control in the presence of FFA or not (Fig. 1C). These results indicate that atRA increases autophagic flux in Hepa1c1c7 cells. To reveal the effect of atRA-induced autophagy on lipid deposition by FFA treatment, we used fatty Hepa1c1c7 cells with knockdown of *Atg5* induced by *Atg5*-specific siRNA. As expected, *Atg5*-specific siRNA reduced the endogenous ATG5 protein levels by more than 60% in fatty Hepa1c1c7 cells (Fig. 1D). *Atg5*-knockdown partly inhibited the atRA-induced increase of the NEFA release from LDs in fatty Hepa1c1c7 cells (Fig. 1E). Taken together, atRA could partially induce lipolysis by enhancing autophagy in fatty Hepa1c1c7 cells.

### atRA induces lipophagy through regulating Rubicon expression in fatty Hepa1c1c7 cells

**Fig. 2:**
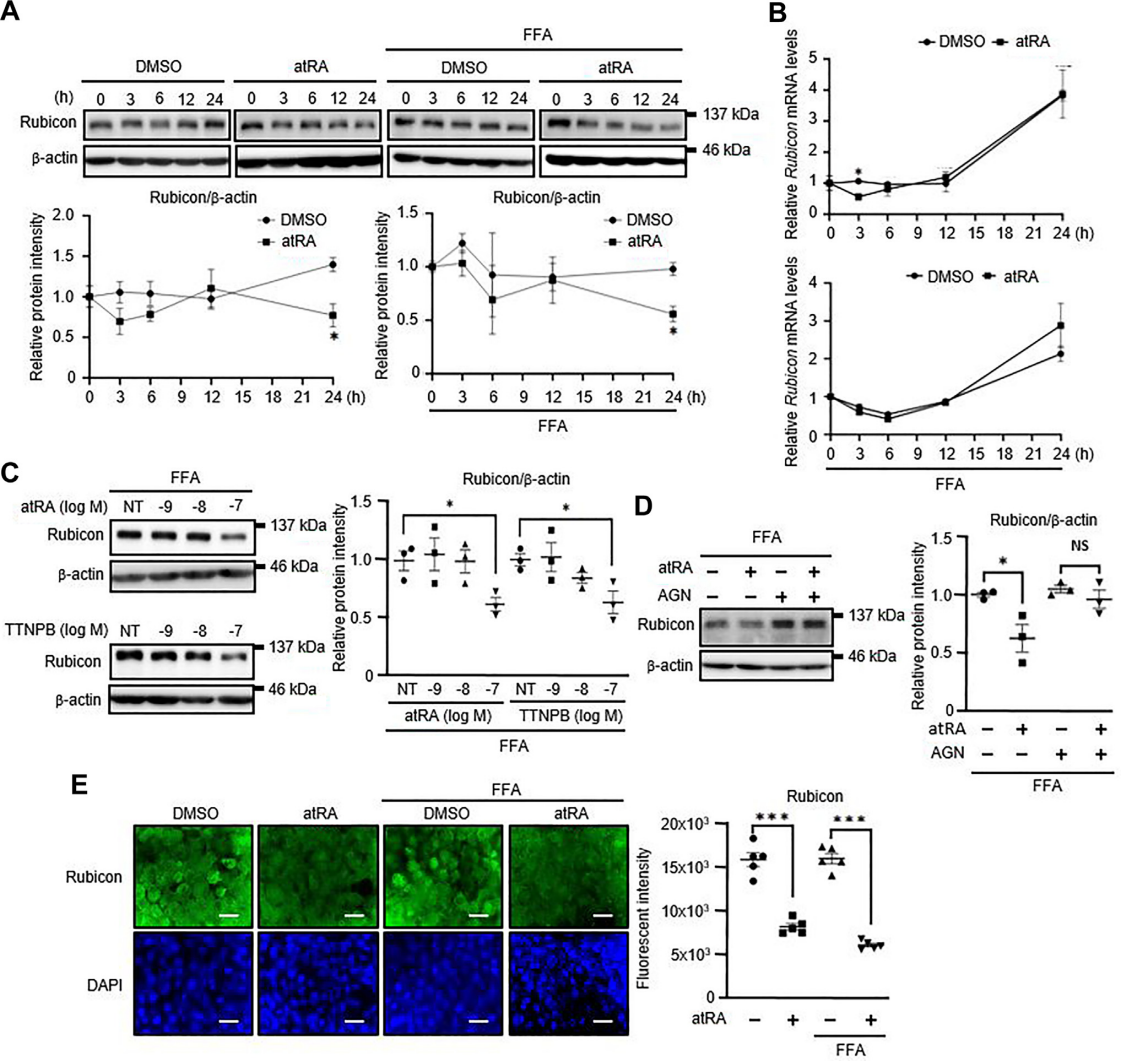
Effects of atRA on the Rubicon expression in Hepa1c1c7 cells. A and B: Hepa1c1c7 or fatty Hepa1c1c7 cells were treated with 100 nM atRA or 0.1% DMSO at the indicated time (0, 3, 6, 12, and 24 h). A: The Rubicon protein was detected by immunoblot analysis with a specific antibody. B: The *Rubicon* mRNA levels were evaluated by real-time PCR. ∗*P* < 0.05 versus DMSO (two-tailed unpaired *t* test). C: Western blotting of Rubicon in fatty Hepa1c1c7 cells treated with vehicle (NT, DMSO) or the indicated concentrations (−9, −8, −7 log M) of atRA or TTNPB for 24 h. ∗*P* < 0.05 versus NT (one-way ANOVA with a Student-Newman *post hoc* test). D: Western blotting of Rubicon in fatty Hepa1c1c7 cells treated by 100 nM atRA with or without 10 μM AGN139109 (AGN) for 24 h. E: Rubicon Ab and a secondary Ab conjugated to Alexa Fluor 488 (green). Nuclear staining with DAPI is shown in blue. Images were taken with a fluorescence microscope. Scale bar = 100 μm. Values are mean ± SEM. (n = 3–4). ∗∗∗*P* < 0.001 (one-way ANOVA with a Student-Newman *post hoc* test).

Rubicon suppresses autophagy at the autophagosome-lysosome fusion step (15, 16, 17). We have previously reported that atRA decreased the Rubicon protein expression in differentiated 3T3-L1 adipocytes (30). To examine the mechanism of how atRA induces autophagy in Hepa1c1c7 cells, we investigated the time-dependent effects of atRA on the Rubicon protein expression in Hepa1c1c7 cells treated with FFA or not. Hepa1c1c7 cells were treated with 100 nM atRA for up to 24 h. Rubicon protein expression significantly decreased at 24 h after atRA treatment in the presence of FFA or not (Fig. 2A). However, the expression of *Rubicon* mRNA was unchanged by atRA treatment in Hepa1c1c7 or fatty Hepa1c1c7 cells (Fig. 2B). We also examine the dose-dependent effects of atRA or TTNPB, a primary agonist of the retinoic acid receptor (RAR), on the decrease of Rubicon protein expression in fatty Hepa1c1c7 cells. Unlike low dose (−9 and −8 log M), a high dose (−7 log M) of atRA or TTNPB significantly decreased Rubicon protein expression in fatty Hepa1c1c7 cells (Fig. 2C). Conversely, AGN139109, a potent pan-RAR antagonist, inhibited atRA-induced reduction of Rubicon expression in fatty Hepa1c1c7 cells (Fig. 2D). In addition, immunofluorescence staining showed that Rubicon expression decreased by atRA in Hepa1c1c7 or fatty Hepa1c1c7 cells (Fig. 2E).

**Fig. 3:**
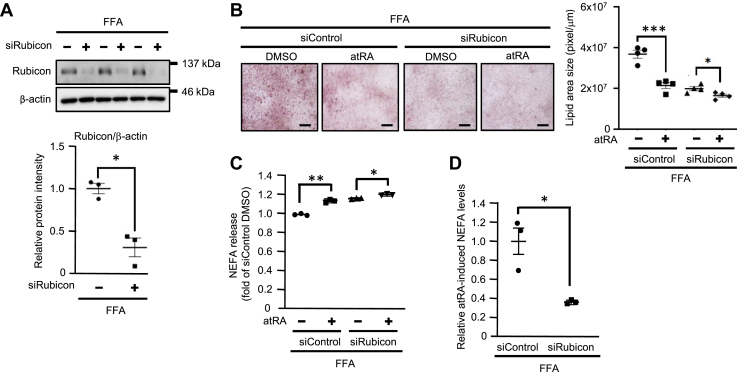
Effects of atRA on the lipophagy via decreasing Rubicon expression in Hepa1c1c7 cells. A: Endogenous Rubicon protein was detected by western blotting 48 h after transfection with 100 pmol Rubicon siRNA (siRubicon) or siControl (n = 3). ∗∗*P* < 0.01 (two-tailed unpaired *t* test). B: Fatty Hepa1c1c7 cells transfected with 100 pmol siRubicon or siControl were treated with 100 nM atRA or 0.1% DMSO for 14 days (n = 3–4). Lipid deposition was stained with oil red-O staining. Scale bar = 100 μm. C and D: fatty Hepa1c1c7 were treated with atRA or DMSO containing 2% BSA-FFA for 48 h after transfection with siRubicon or siControl. The culture medium was collected and assayed for NEFA content (mEq/L/mg protein). The comparison of atRA-induced NEFA levels. Values are mean ± SEM. (n = 3–4). ∗*P* < 0.05, ∗∗*P* < 0.01, ∗∗∗*P* < 0.001 (one-way ANOVA with a Student-Newman *post hoc* test).

Next, to clarify whether Rubicon is associated with lipophagy by atRA, we used fatty Hepa1c1c7 cells with knockdown of *Rubicon* by *Rubicon*-specific siRNA. As expected, *Rubicon*-specific siRNA reduced the endogenous Rubicon protein levels by more than 60% in fatty Hepa1c1c7 cells (Fig. 3A). Oil red-O staining showed that *Rubicon*-knockdown inhibited the atRA-induced decrease in lipid deposition in fatty Hepa1c1c7 cells (Fig. 3B). In addition, we confirmed that *Rubicon*-knockdown significantly inhibited the atRA-induced increase of the release of NEFA from LDs in fatty Hepa1c1c7 cells (Fig. 3C, D). These results suggest that atRA partially induces lipophagy by downregulating Rubicon expression in fatty Hepa1c1c7 cells.

### atRA reduces lipid accumulation and Rubicon protein expression in the liver of mice fed an HFD

**Fig. 4:**
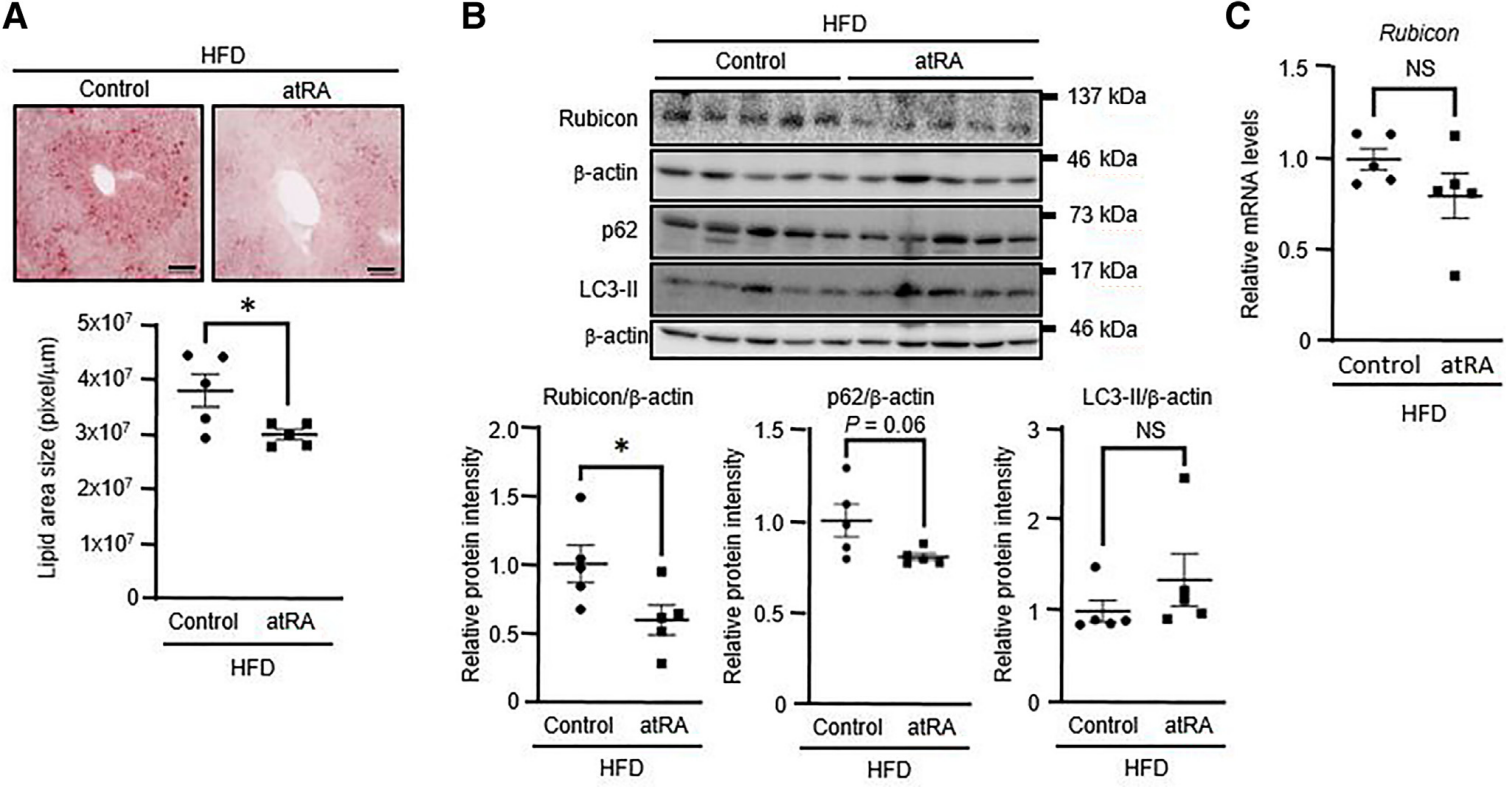
Effects of atRA on the Rubicon expression in the liver of mice fed an HFD. Eight-week-old male mice were fed a high-fat diet (HFD) for 8 weeks and were randomly divided into two groups and treated with 0.1% DMSO (Control) or atRA (10 mg/kg body weight) for 4 weeks (n = 5). A: Oil red-O staining and quantifying lipid deposition in the liver of mice. Scale bar = 100 μm. Lipid accumulation was quantified using the ImageJ imaging software program. B: Western blotting of Rubicon, p62, and LC3-II protein levels in the liver of mice. β-actin was used as an internal control. C: the *Rubicon* mRNA levels were assessed by real-time PCR. Values are mean ± SEM. ∗*P* < 0.05 (two-tailed unpaired *t* test). NS, not significant.

Because Rubicon increases in association with autophagy impairment in the livers of mice fed an HFD (20), we examined the effect of atRA on the expression of Rubicon in the livers of HFD-fed mice. Mice fed an HFD for 8 weeks were randomly divided into two groups and treated with 0.1% DMSO (Control) or atRA 10 mg/kg body weight for 4 weeks. Oil red-O staining demonstrated that atRA treatment significantly decreased hepatic lipid accumulation in mice fed an HFD (Fig. 4A). Treatment of atRA significantly reduced hepatic Rubicon protein expression in HFD-fed mice (Fig. 4B). However, hepatic *Rubicon* mRNA expression was unchanged in the atRA group as well as in vitro experiments (Fig. 4C). Although LC3-II protein expression was unchanged, atRA treatment reduced p62 protein expression in the livers of HFD-fed mice, but not significantly (*P* = 0.06) (Fig. 4B).

### atRA decreased Rubicon protein expression in the livers of young, but not aged, mice

**Fig. 5:**
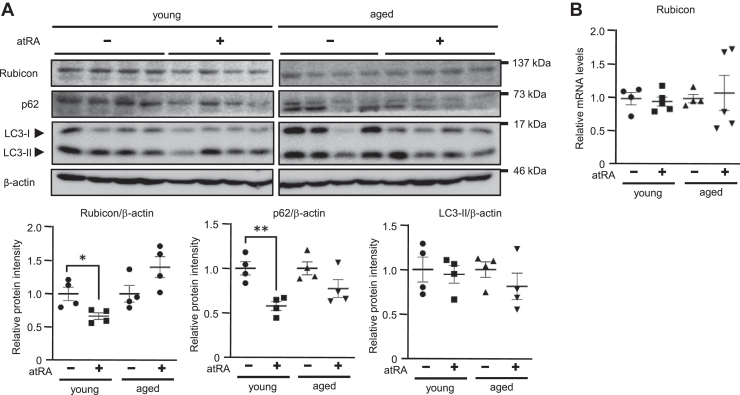
Difference of effects of atRA on the Rubicon expression in the liver of young and aged mice. Eight-week-old and eighty-six-week-old male C57BL/6J mice were randomly divided into two groups and treated with 0.1% DMSO (Control) or atRA (10 mg/kg body weight) for 24 h (n = 4–5). A: Western blotting of Rubicon, LC3-II, and p62 protein levels in the liver of mice. β-actin was used as an internal control. B: the *Rubicon* mRNA levels were assessed by real-time PCR. Values are mean ± SEM. ∗*P* < 0.05, ∗∗*P* < 0.01 (one-way ANOVA with a Student-Newman *post hoc* test).

An age-dependent decline in basal autophagy in the liver may underlie the accumulation of hepatic lipids, which has been proposed to contribute to worsening metabolic conditions and impaired autophagy, resulting in a vicious cycle promoting aging (11, 12). Besides, Rubicon levels increase in association with autophagy impairment in the livers of mice fed an HFD, recapitulating NAFLD (20). In the present study, 8-week-old and 86-week-old mice were treated with 0.1% DMSO (Control) or atRA 10 mg/kg body weight for 24 h to evaluate whether age affects the reduction of Rubicon expression induced by atRA in the liver of mice. Interestingly, although young mice showed that atRA significantly decreased Rubicon and p62 protein levels in the liver, aged mice showed no effects of atRA on their expression (Fig. 5A). In addition, atRA treatment affected no remarkable difference in hepatic *Rubicon* mRNA expression in both young and aged mice (Fig. 5B). Collectively, these findings suggest atRA reduced hepatic Rubicon protein expression in young than aged mice.

### atRA decreases Rubicon protein expression via reducing translation efficiency in Hepa1c1c7 cells

**Fig. 6:**
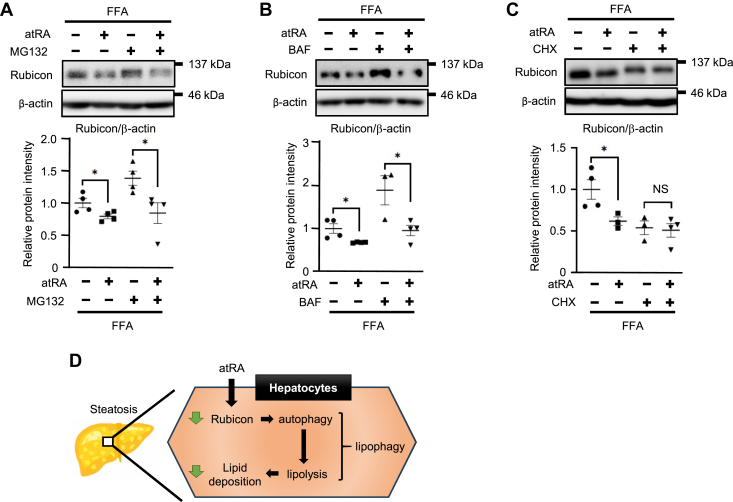
The study of the mechanism by which atRA decreases Rubicon protein expression in Hepa1c1c7 cells. A–C: Western blotting of Rubicon in Hepa1c1c7 or fatty Hepa1c1c7 cells treated by 100 nM atRA with or without (A) 1 μM MG132, (B) 100 nM Bafilomycin A1 (BAF), or (C) 10 μg/ml cycloheximide (CHX) for 24 h. β-actin was used as an internal control (n = 3–4). Values are mean ± SEM. ∗*P* < 0,05, ∗∗*P* < 0,01 (one-way ANOVA with a Student-Newman post hoc test). NS, not significant. D: schematic illustration of the induction of lipophagy via reducing Rubicon expression by atRA in hepatocytes.

Because the above findings suggested that atRA decreased Rubicon protein expression without changing the levels of *Rubicon* mRNA, we hypothesized that atRA increases the degradation of Rubicon protein via proteasome or lysosomal pathway based on recent research (20, 38). Unexpectedly, Western blotting revealed that atRA decreased Rubicon protein levels in the presence of proteasome inhibitor MG132 or lysosomal inhibitor Bafilomycin A1 in fatty Hepa1c1c7 cells (Fig. 6A, B). Therefore, we next hypothesized that atRA might decrease Rubicon protein expression via inhibiting Rubicon protein synthesis. Translation inhibitor cycloheximide blocked the reduction of Rubicon protein by atRA in fatty Hepa1c1c7 cells (Fig. 6C). These results suggested that atRA could down-regulate Rubicon protein expression via decreasing Rubicon protein synthesis in hepatocytes.

## Discussion

In the present study, we determined that atRA contributes to lipolysis via autophagy through down-regulating Rubicon expression in fatty Hepa1c1c7 cells. NAFLD, defined by the accumulation of LDs in hepatocytes, increases the risk of type II diabetes, cardiovascular disease, chronic kidney disease, liver cirrhosis, and hepatocellular carcinoma (1, 2). Because lipophagy can decrease hepatic steatosis and is impaired in NAFLD patients, promoting lipophagy may be a therapeutic approach to treat NAFLD (1, 8). Some studies reported that atRA reduced hepatic lipid accumulation in liver steatosis model animals (28). Recently, although we found that atRA induces lipophagy by activating the AMPK pathway in mice adipocytes (30), it remains unclear whether atRA ameliorates hepatic steatosis by inducing lipophagy. Here, we observed that atRA decreased the FFA-induced lipid accumulation in Hepa1c1c7. We also found that atRA increased autophagic activity in Hepa1c1c7 or fatty Hepa1c1c7 cells from the results of p62 expression and the GFP/mCherry ratio with the GFP-LC3-mCherry probe. In addition, we confirmed that *Atg5* knockdown partially blocked the increased release of NEFA from LDs by atRA in fatty Hepa1c1c7 cells. These results were similar to our previous study, which found that atRA partially contributes to lipolysis through autophagy in 3T3-L1 adipocytes (30). In addition, Wu *et al.* reported that retinoic acid activates autophagy in renal tubular epithelial cells treated with or without cisplatin, an anti-tumor drug (39). These reports suggest that the induction of autophagy by atRA is not liver-specific. In the present study, we confirmed that atRA induces autophagy not only in fatty Hepa1c1c7 cells but also in Hepa1c1c7 cells untreated with fatty acid. Fang *et al.* reported that atRA induces autophagy in mouse and human hepatocarcinoma cells untreated with fatty acid (40). That is to say, atRA activates autophagy in the liver, and just one of the downstream consequences is the release of NEFA.

Two central processes mediate the breakdown of triglycerides stored within LD: cytosolic lipolysis and lipophagy. The classical lipolysis pathway is mediated by three cytosolic lipases, including adipose triglyceride lipase (ATGL), HSL, and monoglyceride lipase (41). Interestingly, LC3, an autophagy substrate, promotes the movement of cytoplasmic ATGL to LD through interaction with the LC3-interacting region domain of ATGL and induces lipophagy (42). In addition, cytosolic lipolysis targets the larger-sized LD to make them smaller, and then lipophagy targets the smaller-sized LD (43). These reports suggest that cytosolic lipolysis and lipophagy are not independent but operate in tandem. atRA has also been reported to reduce hepatic lipid accumulation in liver steatosis model animals by repressing PPARγ and to induce lipolysis by a PPARβ/δ-mediated increase in the levels of HSL in adipocytes (28, 29). These results and our findings suggest that atRA reduces the lipid accumulation in hepatocytes by enhancing lipophagy, cytosolic lipolysis in conjugation with autophagy, and HSL and lowering lipogenesis.

Generally, autophagy can be induced in cells by activating the AMPK pathway or reducing the expression of Rubicon, a well-known negative regulator of autophagy (13, 14, 15, 16, 17). Interestingly, Rubicon expression increased in the livers of mice fed an HFD, and hepatocyte-specific *Rubicon* knockout improved the autophagy activity and liver steatosis in mice fed an HFD (20). Therefore, Rubicon is a potential target for the treatment of NAFLD. Several studies have reported aging, palmitic acid, and hepatitis B or C virus as positive factors for hepatic Rubicon expression (18, 20, 44, 45), but not much has been reported on negative regulators. Recently, we have presented that atRA reduces Rubicon protein expression in adipocytes, but we have not demonstrated whether Rubicon is associated with the induction of lipophagy by atRA in adipocytes (30). Here, we also showed that treating atRA or TTNPB, a primary agonist of RAR, decreased Rubicon protein expression in fatty Hepa1c1c7 cells. Conversely, a potent pan-RAR antagonist inhibited atRA-induced reduction of Rubicon expression in fatty Hepa1c1c7 cells. Besides, *Rubicon* knockdown could partially block the reduced lipid deposition by atRA and increase the release of NEFA from LDs by atRA in fatty Hepa1c1c7 cells. These results indicated that atRA induces lipophagy via decreasing Rubicon protein levels in mice hepatocytes. In addition, Minami *et al.* recently demonstrated that liver lipophagy prevents liver steatosis in mice fed an HFD (46). Taken together, atRA may be effective as a treatment strategy for NAFLD. However, further studies are necessary to fully elucidate the underlying molecular mechanism of atRA on the lipophagy via Rubicon in the liver and the adverse effects on the body.

The present study confirmed that atRA reduces Rubicon protein expression without altering *Rubicon* mRNA levels in fatty Hepa1c1c7 cells. These results were similar to our previous results in adipocytes (30). As mentioned above, hepatic Rubicon expression increases in the mice fed an HFD or aged mice (18, 20). The present study confirmed that atRA reduced hepatic Rubicon expression in the mice fed an HFD. In addition, atRA decreased Rubicon expression in the livers of young mice, but the effect of atRA on reduced Rubicon expression diminished in the livers of aged mice. The reason may be that the mechanism by which an HFD increases Rubicon expression differs from the mechanism by which aging increases it (20, 38). Tanaka *et al*. reported that palmitic acid upregulates hepatic Rubicon expression by decreasing degradation via the proteasome system (20). In contrast, Yamamuro *et al*. reported that aging increases Rubicon expression in adipocytes by decreased degradation via the autophagy pathway (38). Indeed, autophagic activity decreases with age in many species (47). However, experiments using proteasome or lysosomal inhibitors confirmed that atRA decreased Rubicon protein levels in a pathway distinct from these pathways in fatty Hepa1c1c7 cells. Therefore, we hypothesized that atRA affects Rubicon protein synthesis but not Rubicon protein degradation. Our study suggested that atRA decreases Rubicon expression by inhibiting protein synthesis in fatty Hepa1c1c7 cells by experiment using a translation inhibitor. The major signaling protein complex that regulates mRNA translation is mTORC1. Activated mTORC1 promotes mRNA translation by phosphorylating various proteins, including eukaryotic initiation factor 4E (eIF4E)/eIF4E-binding protein 1 (4E-BP1) and S6 kinase. Phosphorylation of 4E-BP1 by mTORC1 blocks the interaction between eIF4E and 4E-BP1; therefore, eIF4E can participate in the eIF4F complex (consists of eIF4G, eIF4A, and eIF4E) to initiate translation (48, 49). Interestingly, atRA binding to RARγ inhibits protein synthesis of myoblast determination protein (MyoD) by the dephosphorylation of 4E-BP1 (50). Therefore, although the effect of atRA on the reduced Rubicon expression may be dependent on protein synthesis in hepatocytes, we have not determined the detailed molecular mechanism of it.

In conclusion, as shown in Fig. 6D, the present study suggested that atRA induces lipophagy by decreasing Rubicon protein in hepatocytes. We also determined that atRA ameliorates lipid accumulation in the livers and decreases hepatic Rubicon expression in mice fed an HFD. In addition, our study demonstrated that atRA reduces hepatic Rubicon protein in young mice, but the ability of atRA to reduce hepatic Rubicon expression diminished in aged mice.

## Data Availability

All data are contained within the manuscript.

## Supplemental data

This article contains supplemental data.

## Conflict of interest

The authors declare that they have no conflicts of interest with the contents of this article.
