# Supplementary material for: All-trans retinoic acid induces lipophagy by reducing Rubicon in Hepa1c1c7 cells
Source: J Lipid Res. 2024 Jul 18;65(8):100598. doi: 10.1016/j.jlr.2024.100598 (PMC11381443; doi:10.1016/j.jlr.2024.100598)
Supplement: Supplemental Data [file mmc1.pdf]

Supplementary Figure 1. Nguyen *et al*.

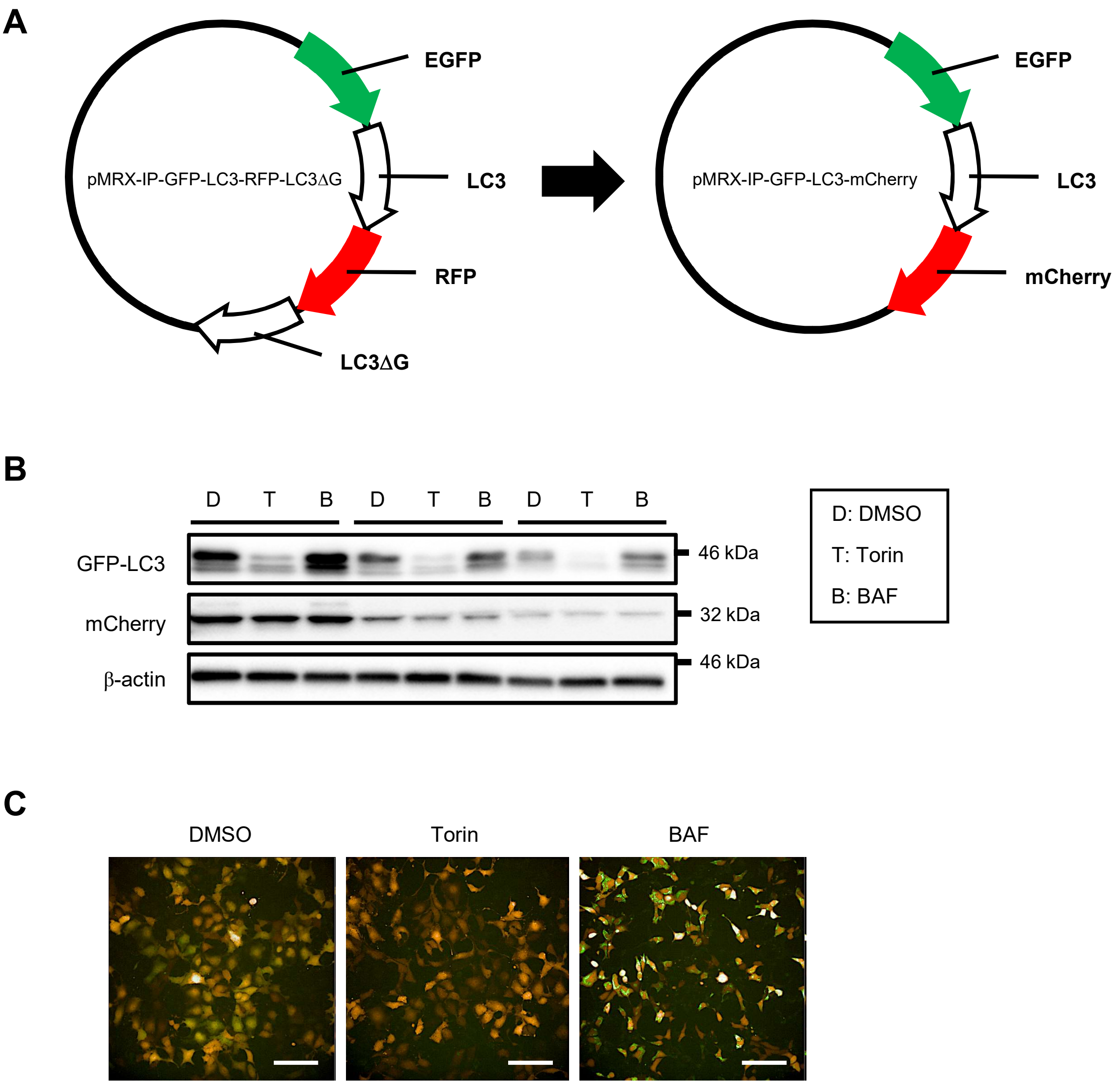

Figure S1. Retroviral plasmid pMRX-IP-GFP-LC3-mCherry. (A) Basic vector map for the original pMRX-IP-GFP-LC3-RFP-LC3ΔG vector and our modified pMRX-IP-GFP-LC3-mCherry vector. (B) Western blotting of GFP-LC3 and mCherry proteins in Hepa1c1c7 cells stably expressing GFP-LC3-mCherry treated with 250 nM Torin, 100 nM Bafilomycin A1 (BAF) or DMSO (vehicle) for 12 h (n = 3). β-actin was used as an internal control. (C) Autophagic flux in Hepa1c1c7 cells stably expressing GFP-LC3-mCherry treated with 250 nM Torin, 100 nM Bafilomycin A1 (BAF) or DMSO (vehicle) for 12 h. Images were taken with a fluorescence microscope. Scale bar = 50 µm.
